# Combination of Paeoniae Radix and Cnidii Rhizoma Prolonged Survival of Fully Mismatched Cardiac Allografts and Generated Regulatory Cells in Mice

**DOI:** 10.1155/2014/841408

**Published:** 2014-03-20

**Authors:** Xiangyuan Jin, Lei Yu, Masateru Uchiyama, Enzhi Yin, Tadanori Harada, Ken Otsuka, Shigefumi Matsuyama, Tomohiro Imazuru, Tomoki Shimokawa, Masanori Niimi

**Affiliations:** ^1^Department of Surgery, Teikyo University, 2-11-1 Kaga, Itabashi-ku, Tokyo 173-8605, Japan; ^2^Department of Cardiovascular and Thoracic Surgery, the 4th Affiliated Hospital of Harbin Medical University, 37 Yiyuan Street, Nangang, Harbin, Heilongjiang 150001, China; ^3^Department of General Surgery, the 2nd Affiliated Hospital of Harbin Medical University, 246 Xuefu Road, Nangang, Harbin, Heilongjiang 150086, China; ^4^Department of Cardiovascular Surgery, Teikyo University, 2-11-1 Kaga, Itabashi-ku, Tokyo 173-8605, Japan; ^5^Department of Cardiovascular Surgery, the 2nd Affiliated Hospital of Harbin Medical University, 246 Xuefu Road, Nangang, Harbin, Heilongjiang 150086, China

## Abstract

In previous studies, we have demonstrated that Tokishakuyakusan (TJ-23) can prolong the survival of allogeneic cardiac grafts and induce regulatory T cells. In this study we investigated the effects of Paeoniae radix and Cnidii rhizoma, two components of TJ-23, on alloimmune responses in a murine cardiac transplantation model and whether the two agents have synergistic effect. CBA mice underwent transplantation of a C57BL/6 heart and received oral administration of 2 g/kg/day of Paeoniae radix, Cnidii rhizoma, or the mixture of two agents from the day of transplantation until 7 days afterward. Naïve CBA mice rejected C57BL/6 cardiac graft acutely (median survival time (MST): 7 days). Paeoniae radix and Cnidii rhizoma prolonged C57BL/6 allograft survival (MSTs: 13.5 and 15.5 days, resp.). However, the mixture of two agents prolonged C57BL/6 allograft survival indefinitely (MST > 100 days). Secondary CBA recipients given whole splenocytes from primary combination-treated CBA recipients with B6 cardiac allografts 30 days after grafting had prolonged survival of B6 hearts (MST: 33 days). Flow cytometry studies showed that the CD4^+^CD25^+^Foxp3^+^ regulatory cell population was increased in combination-treated recipients. Combination of Paeoniae radix and Cnidii rhizoma induced hyporesponsiveness to fully allogeneic cardiac allografts and may generate CD4^+^CD25^+^Foxp3^+^ regulatory cells in our model.

## 1. Introduction

Since Japanese government health officials officially recognized the therapeutic effects of Japanese herbal (Kampo) medicines about 30 years ago, these medicines have been widely used as alternative therapy for several diseases. In recent studies of our murine model, oral administration of the Japanese herbal medicines Tokishakuyakusan (TJ-23) [1], Saireito (TJ-114) [2], or Artemisiae Capillaris Herba [3] was associated with significantly prolonged survival of allogeneic cardiac grafts (median survival times (MSTs) of all groups > 100 days) and generation of regulatory cells. Paeoniae radix (*Paeonia lactiflora*) and Cnidii rhizoma (*Cnidium officinale*) were two components of TJ-23, and, in this study, we examined whether the combination of Paeoniae radix and Cnidii rhizoma could affect the duration of allograft survival in the same model.

## 2. Materials and Methods

### 2.1. Animals

Male C57BL/6 (B6, H2^b^), CBA (H2^k^) mice that were 8–12 weeks were purchased from Sankyo Ltd. (Tokyo, Japan), housed in conventional facilities at the Biomedical Services Unit in Teikyo University, and used in accordance with the guidelines for animal experimentation approved by the Animal Use and Care Committee of Teikyo University, and used in accordance with the guidelines for animal experimentation approved by the Animal Use and Care Committee of the university and the* Principles of Laboratory Animal Care* (NIH publication, vol. 25, no. 28, revised 1996).

### 2.2. Heart Transplantation

All transplant procedures were performed on the mice under general anesthesia. Fully vascularized abdominal heterotopic hearts from B6 donors were transplanted into CBA mice by microsurgical techniques [4]. Postoperatively, graft function was assessed daily by palpation for evidence of contraction. Rejection was defined as complete cessation of the heart beat and confirmed by direct visualization and histologic examination of the graft.

### 2.3. Treatment with Japanese Herbal Medicines

Naïve CBA recipients of a B6 heart were given no treatment, distilled water (control group), or oral administration of 2 g/kg/day of each component of TJ-23 (Poria sclerotium, Cnidii rhizoma, Paeoniae radix, Atractylodis lanceae rhizoma, Alismatis rhizoma, and Angelicae radix) or mixture of two components (Poria sclerotium, Cnidii rhizoma, and Paeoniae radix) from the day of transplantation to 7 days afterward. Other CBA recipients were also given mixture of 0.2 g/kg/day of Paeoniae radix and Cnidii rhizoma or mixture of 0.02 g/kg/day of Paeoniae radix and Cnidii rhizoma. All the agents were dissolved in distilled water and given orally by a metal tube (Thomas Scientific, Swedesboro, NJ). The agents were made by boiling water extraction, separating the effluents from the residuals, and spray-drying the effluents to produce the extract powder [5]. All the agents were made as frozen dry powder gifted by Tsumura (Tokyo, Japan).

### 2.4. Adoptive Transfer Studies

Adoptive transfer studies were conducted to determine whether regulatory cells were generated after treatment with mixture of Paeoniae radix and Cnidii rhizoma. Thus, 30 days after CBA recipients (primary recipients) underwent transplantation of a B6 cardiac allograft and were given mixture of Paeoniae radix and Cnidii rhizoma, splenocytes (5.0 × 10^7^) from primary recipients with functioning allografts were adoptively transferred into naive CBA mice (secondary recipients). After the adoptive transfer, the secondary recipients underwent transplantation of a B6 heart immediately.

### 2.5. Histologic Studies of Harvested Grafts

Cardiac allografts in untreated mice and mice given mixture of Paeoniae radix and Cnidii rhizoma were removed 14 days after transplantation and studied histologically and immunohistochemically. Histologic and immunohistochemical staining were performed as described previously [6]. Hematoxylin and eosin (HE) staining was assessed by grading with a semiquantitative scale for the amount of mononuclear cell infiltration (0, no infiltration; 1, faint and limited infiltration; 2, moderate infiltration; 3, severe infiltration) [7, 8]. In immunohistochemical (IHC) study, the number of infiltrating Foxp3^+^ cell in mixture-treated or untreated mice was counted. All graft heart slides were assessed blindly by unrelated one researcher.

### 2.6. Flow Cytometry Analysis

The expression of CD4, CD25, and Foxp3 in splenocytes was determined by flow cytometry. After cardiac allograft transplantation for 14 days, splenocytes from CBA recipients given mixture of Paeoniae radix and Cnidii rhizoma, untreated recipients, and naïve CBA with/without mixture of Paeoniae radix and Cnidii rhizoma were stained with fluorochrome-conjugated anti-CD4 or anti-CD25 monoclonal antibody (mAb) (RM4-5 and PC61, resp.; BD Biosciences, San Jose, CA, USA) and anti-mouse Foxp3 mAb (FJK-16s; eBioscience, San Diego, CA), as well as their isotype controls (eBioscience). The stained cells were analyzed by using a FACS Canto2 system (BD Biosciences). The percentage of CD4^+^ CD25^+^ Foxp3^+^ in CD4^+^ cells was determined.

### 2.7. Mixed Leukocyte Culture and Cytokine Assays

In mixed leukocyte culture (MLC) studies [9], the responder cells were splenocytes from naïve CBA mice, untreated, Paeoniae radix-treated, Cnidii rhizoma-treated, or Paeoniae radix and Cnidii rhizoma-treated CBA mice that had undergone transplantation of a B6 heart 14 days earlier. The stimulator cells were B6 (allogeneic) splenocytes treated with 100 *μ*g/mL mitomycin C (MMC) (Kyowa Hakko, Osaka, Japan) for 30 minutes at 37°C. The responder cells (2.5 × 10^6^/mL) were cocultured with the stimulator cells (5.0 × 10^6^/mL) in complete medium in a humidified 5% CO_2_ atmosphere (CH-16M, Hitachi, Tokyo, Japan) at 37°C in 96-well, round-bottomed tissue culture plates (Iwaki Scitech Division, Tokyo, Japan) for 4 days. Proliferation was assessed by using an enzyme-linked Immunosorbent assay (ELISA) for bromodeoxyuridine incorporation (Biotrak, version 2, Amersham, Little Chalfont, UK) according to the manufacturer's instructions.

In some experiments, the MLC contained splenocytes from naïve CBA (responder cells; 2.5 × 10^6^/mL) and MMC-treated splenocytes from B6 (stimulator cells; 5 × 10^6^/mL). Two amounts of the mixture (0.5 and 5 mg/mL) were added to the MLC to assess the direct effects of these agents on cellular proliferation (direct MLC).

An ELISA was also performed to assess levels of IL-2, IL-4, IL-10, and interferon (IFN)-*γ* in the supernatant of the MLC on day 4. The capture mAb (JES5-2A5), detection mAb (JES5-16E3), and recombinant standard for IL-10 were from BD Biosciences. The capture and detection mAbs for IL-2 (JES6-1A12 and JES6-5H4, resp.), IL-4 (BVD-1D11 and BVD-24G2, resp.), and IFN-*γ* (R4-6A2 and XMG1.2, resp.) were from Caltag Laboratories (Burlingame, CA). Recombinant standards for IL-2, IL-4, and IFN-*γ* were from PeproTech (London, UK).

### 2.8. Statistical Analysis

Cardiac allograft survival in groups of mice was compared by using Mann-Whitney *U* testing (Graphpad Prism; Graphpad, CA). In the cell-proliferation, cytokine, and flow cytometry studies and the difference between two groups was assessed using an unpaired Student's *t*-test or analysis of variance (ANOVA) with Ryan method. A value of *P* < 0.05 was considered statistically significant.

## 3. Results

### 3.1. Survival of Cardiac Allografts in Mice Treated with Agents

**Figure 1:**
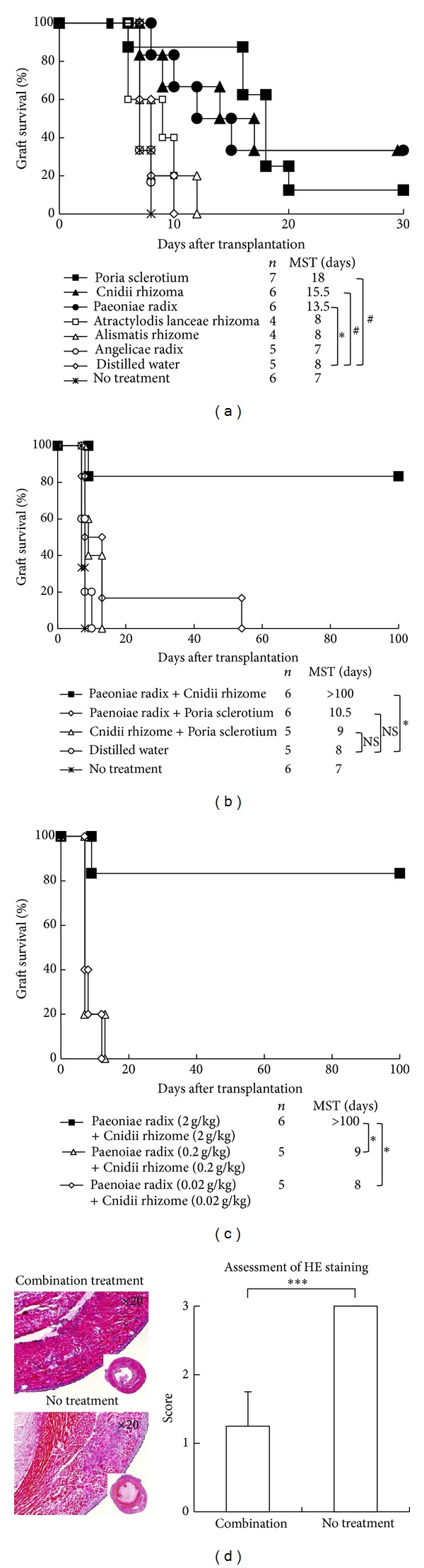
Graft survival of CBA mice given oral administration of agents and histologic studies. (a) Recipients with C57BL/6 hearts were either untreated, given distilled water, or given 2 g/kg/day of TJ-23 from the day of transplantation until 7 days afterward. MST: median survival time; ^#^ *P* < 0.05 and *P < 0.01 for difference between 2 groups. (b) CBA recipients with C57BL/6 hearts were treated with mixture of two agents (2 g/kg/day of Poria sclerotium, Cnidii rhizoma, and Paeoniae radix) from the day of transplantation to 7 days afterward. MST, median survival time; *P < 0.01 for difference between 2 groups; NS = no significant difference. (c) CBA recipients with C57BL/6 hearts were treated with various amounts of mixtures of Paeoniae radix and Cnidii rhizoma. MST: median survival time; *P < 0.01 for difference between 2 groups. (d) Histologic studies of harvested cardiac allografts stained with hematoxylin-eosin (HE). The upper left picture shows a representative sample obtained from mice given mixture of Paeoniae radix and Cnidii rhizoma and the lower left picture shows the sample from untreated mice (magnification ×20 of two pictures). The right graph shows the degree of mononuclear cell infiltration was assessed by grading with semiquantitative scale (0, no infiltration; 1, faint and limited infiltration; 2, moderate infiltration; 3, severe infiltration). ***P < 0.001 for difference between 2 groups.

CBA recipients without any treatment or given distilled water rejected B6 grafts acutely (median survival times (MSTs): 7 and 8 days, resp.; Figure 1(a)). When CBA recipients were given 2 g/kg/day of each component of TJ-23, only Poria sclerotium, Cnidii rhizoma, and Paeoniae radix had prolonged B6 allograft survival (MSTs: 18, 15.5 and 13.5 days, resp.; *P* < 0.05, < 0.05, and < 0.01 versus distilled water group, resp.). Moreover, when CBA recipients received mixture of Paeoniae radix and Cnidii rhizoma, Paeoniae radix, and Poria sclerotium or Cnidii rhizoma and Poria sclerotium, only the combination of Paeoniae radix and Cnidii rhizoma prolonged B6 allograft survival indefinitely (MST >100 days; *P* < 0.01 versus distilled water group; Figure 1(b)).

When CBA recipients were given mixture of 0.2 g/kg of Paeoniae radix and Cnidii rhizoma or mixture of 0.02 g/kg/day of Paeoniae radix and Cnidii rhizoma, neither of them could prolong the survival of B6 cardiac allograft (Figure 1(c)). We did not find any severe immune suppressive side effect (such as infections and tumor) of the mixture in this study.

### 3.2. Histologic Features of Allografts from Recipients Treated with Mixture of Paeoniae Radix and Cnidii Rhizome

Histologic examinations of cardiac allografts obtained 14 days after transplantation showed cell infiltrated but significantly preserved structure with a few myocardial injuries in transplant recipients given mixture of Paeoniae radix and Cnidii rhizoma, whereas allografts from untreated recipients showed severe myocyte damage and edema of the acute rejection process (Figure 1(d)). Moreover, in each section of HE staining there was a significant difference by grading with a semiquantitative scale [6, 7].

### 3.3. Generation of Regulatory Cells in Mice Treated with Agents

**Figure 2:**
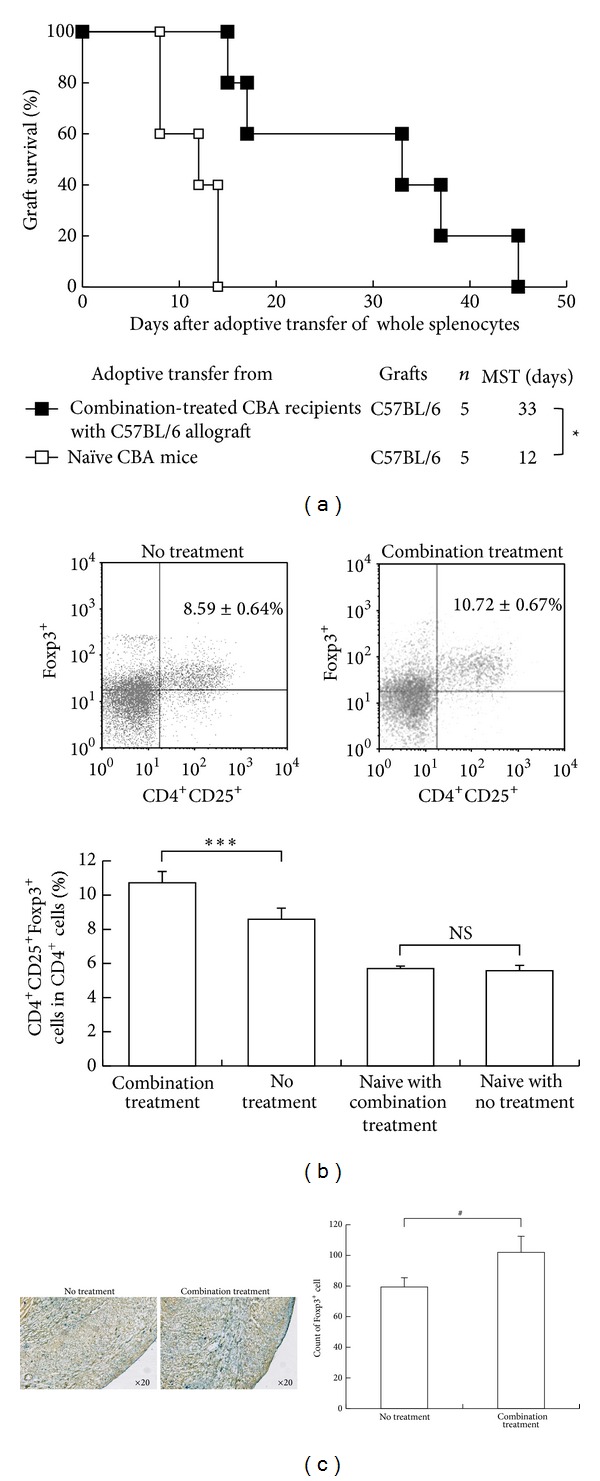
Evidence of generation of regulatory cells in CBA allograft recipients treated with combination of Paeoniae radix and Cnidii rhizoma. (a) Cardiac allograft survival after adoptive transfer of whole splenocytes. MST: median survival time; *P < 0.01 for difference between 2 groups. (b) CD4, CD25, and Foxp3 expression in splenocytes, as determined by flow cytometry. The upper panels show representative data of dot plots on flow cytometry and the percentage of CD4^+^CD25^+^Foxp3^+^ in CD4^+^ cells from splenocytes in no treatment and combination of Paeoniae radix and Cnidii rhizoma. Data are mean ± SD (*n* = 5 mice in each group). ***P < 0.001 for difference between 2 groups. (c) Infiltration of Foxp3^+^ cells in cardiac grafts 14 days after grafting from untreated or mixture-treated recipients (magnification ×20). The right graph shows the count of infiltrating Foxp3^+^ cells in cardiac allografts form untreated or mixture-treated recipients. ^#^ *P* < 0.05 for difference between 2 groups.

In the adoptive transfer study, secondary CBA recipients given whole splenocytes from primary mixture of Paeoniae radix and Cnidii rhizoma-treated CBA recipients with B6 cardiac allografts 30 days after grafting had significantly prolonged survival of B6 hearts compared to secondary recipients which were adoptively transferred from naïve CBA splenocytes (MSTs: 33 days and 12 days, resp.; *P* < 0.01; Figure 2(a)).

In flow cytometry study, the percentage of population of CD4^+^CD25^+^Foxp3^+^ cells in CD4^+^ cells was increased in the spleens of recipients given mixture of Paeoniae radix and Cnidii rhizoma compared with those of untreated CBA recipients (*P* < 0.001; Figure 2(b)). There is no significant difference in the percentage of population of CD4^+^CD25^+^Foxp3^+^ cells in CD4^+^ cells between the naïve CBA with and without mixture of Paeoniae and Cnidii (Figure 2(b)). Additionally, IHC study showed the number of Foxp3^+^ cells in cardiac grafts from mixture-treated CBA recipients obviously increased more than in untreated recipients (*P* < 0.05; Figure 2(c)).

### 3.4. Cell Proliferation and Cytokine Production in Mice

**Figure 3:**
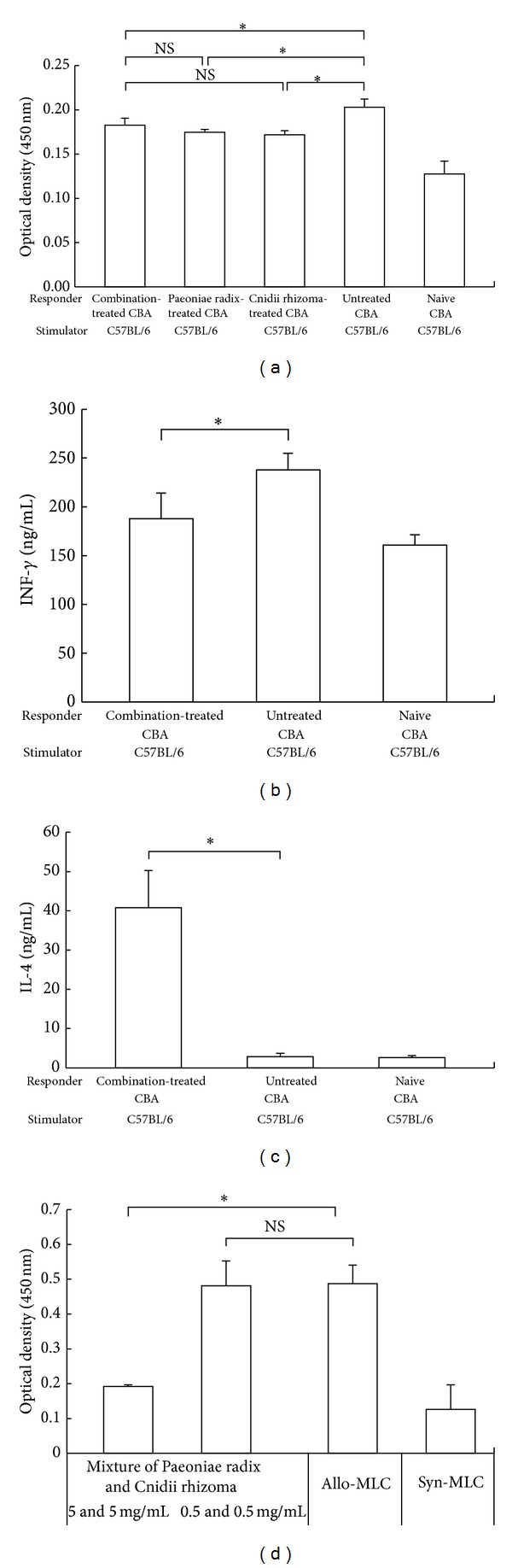
Evidence of induction of alloproliferative hyporesponsiveness in CBA recipients of allograft treated with combination of Paeoniae radix and Cnidii rhizoma. (a) Results of cell-proliferation assays in mixed leukocyte cultures (MLCs). The data shown are mean ± SD values derived from samples from 6 mice in each group. *P < 0.01 for difference between 2 groups. NS: no significant difference. (b) and (c) Levels of cytokines in MLCs. Levels of interferon-*γ* (b) and interleukin 4 (IL-4) (c) in the MLCs were assessed by enzyme-linked immunosorbent assays. Data are shown as mean ± SD values derived from samples from 6 mice in each group. *P < 0.01 for difference between 2 groups. (d) Direct effect of combination of Paeoniae radix and Cnidii rhizoma on alloproliferation in MLC. The data shown are mean ± SD values derived from samples from 6 mice in each group. *P < 0.01 for difference between 2 groups. NS: no significant difference.

Proliferation of splenocytes from CBA recipients were significantly suppressed in treatment with Paeoniae radix, Cnidii rhizoma, and mixture of Paeoniae radix and Cnidii rhizoma compared with that of splenocytes from untreated mice (each *P* < 0.01; Figure 3(a)).

Level of IFN-*γ* was decreased (*P* < 0.01; Figure 3(b)) and that of IL-4 was increased (*P* < 0.01; Figure 3(c)) in splenocytes from CBA recipients treated with mixture of Paeoniae radix and Cnidii rhizoma compared with untreated CBA mice. There was no difference between the two groups in levels of IL-2 and IL-10 (data not shown).

Moreover, the addition of mixture of Paeoniae radix and Cnidii rhizoma to an allogeneic MLC inhibited proliferation of CBA responder cells against B6 stimulator cells in a dose-dependent manner (Figure 3(d)).

## 4. Discussion

There are several mechanisms by which treatment with combination of Paeoniae radix and Cnidii rhizoma has induced increased allograft survival in our model. The first possible mechanism of our results is the generation of regulatory cells. Active suppression by regulatory cells has been found to be one of the important mechanisms for induction and maintenance of self-tolerance [10] and unresponsiveness to allografts [11] and prevention of vasculopathy in cardiac allografts [12–16]. Additionally, many manuscripts on herbal medicines we have ever submitted indicated the potential of immunomodulation and immunosuppressive effect of herbal medicines [1, 2]. Also, one recent study showed triptolide, one component of Chinese herbal medicine, facilitated the expansion of regulatory T cells via modulation of dendritic cells [17]. In our adoptive transfer study (Figure 2(a)), naïve secondary CBA recipients given whole splenocytes from combination-treated primary CBA recipients with functioning B6 cardiac allografts had significant prolongation of allograft survival of their B6 cardiac allograft. In addition, our current studies showed that the population of CD4^+^CD25^+^Foxp3^+^ cells in the spleens clearly increased in flow cytometry (Figure 2(b)) and mixture-treated recipients visibly had more Foxp3^+^ cells than untreated recipients (Figure 2(c)). Moreover, the alloproliferation of splenocytes from Paeoniae radix and Cnidii rhizoma-treated CBA recipients was markedly suppressed in MLC (Figure 3(a)). These data suggest that treatment with combination of Paeoniae radix and Cnidii rhizoma generated regulatory cells in primary CBA recipients.

A second possible mechanism for combination of Paeoniae radix and Cnidii rhizoma-induced hyporesponsiveness in our model is that the balance between Th-1 and Th-2 cytokines secretion may have a strong influence on the function of regulatory cells. Our previous studies have demonstrated that inducible regulatory T cells might enable the change of Th-1/Th-2 cytokines [6, 18]. Moreover, some studies demonstrated that administration of traditional herbal medicines could change Th-1/Th2 cytokines in mice [19, 20]. In our current study, a notable increase of Th-2 cytokine (IL-4) and a decrease of Th-2 cytokine (IFN-*γ*) were detected in the Paeoniae radix and Cnidii rhizoma-treated CBA recipients (Figures 3(b) and 3(c)), suggesting that administration of Paeoniae radix and Cnidii rhizoma might generate regulatory cells via Th-1/Th-2 cytokine changes.

A third possible mechanism is the immunosuppressive effects by mixture of Paeoniae radix and Cnidii rhizoma. Actually, many reports demonstrated that some herbal medicines such as Kakkonto had the potentials of anti-inflammatory to allergy [21]. and one report showed that administration of Paeoniae radix extracts inhibited IL-10 and enhanced IL-8 expression [22]. However, the effects of mixture of Paeoniae radix and Cnidii rhizoma have not been reported. In our model, primary CBA recipient treated with mixture of Paeoniae radix and Cnidii rhizoma had more prolonged allograft survival compared with monotreatment with either Paeoniae radix or Cnidii rhizoma. Additionally, histologic studies of allografts obtained from combination-treated recipients showed much less leukocyte infiltration and maintenance of myocardial structure than those from no treatment recipients (Figure 1(d)), and the addition of mixture of Paeoniae radix and Cnidii rhizoma to an allogeneic MLC inhibited proliferation of CBA responder cells against B6 stimulator cells in a dose-dependent manner (Figure 3(d)). Therefore, our current results indicate that treatment with mixture of Paeoniae radix and Cnidii rhizoma may have the ability to inhibit activation of alloreactive T cells directly or in some other unknown mechanisms.

## 5. Conclusion

These findings demonstrated that treatment with Paeoniae radix and Cnidii rhizoma had an ability that induced prolonged allograft survival and generation of regulatory CD4^+^CD25^+^Foxp3^+^ cells in our* in vivo* model.
